# Mn_3_O_4_ Nanoenzyme Seed Soaking Enhanced Salt Tolerance in Soybean Through Modulating Homeostasis of Reactive Oxygen Species and ATPase Activities

**DOI:** 10.3390/plants13213011

**Published:** 2024-10-28

**Authors:** Tingyong Mao, Linfeng Bao, Hengbin Zhang, Zhilin Shi, Jiahao Liu, Desheng Wang, Chan Liu, Yong Zhan, Yunlong Zhai

**Affiliations:** 1 College of Agriculture, Tarim University, Alar 843300, China; tymao@taru.edu.cn (T.M.); blfbzb@163.com (L.B.); 15993096298@163.com (Z.S.); 13163247758@163.com (J.L.); wds1858@163.com (D.W.); 18634763667@163.com (C.L.); 2 Key Laboratory of Tarim Oasis Agriculture, Ministry of Education, Tarim University, Alar 843300, China; 3 Crops Research Institute, Xinjiang Academy of Agricultural and Reclamation Sciences, Shihezi 832000, China; shzzhhb@163.com

**Keywords:** adenosine triphosphate, soybean, reactive oxygen species homeostasis, nano-enabled agriculture, salinity stress, Mn_3_O_4_ nanoparticles

## Abstract

Soybean, an important cash crop, is often affected by soil salinity, which is one of the important types of abiotic stress that affects its growth. Poly (acrylic) acid coated Mn_3_O_4_ (PMO) has been reported to play a vital role in defending against a variety of abiotic stresses in plants. To date, the effects of PMOs on soybean have not been reported; this study explored the mechanism of PMO-enhanced soybean germination under salt stress. In this experiment, 100 mg/L PMO was used as an immersion agent with a salt treatment of 150 mM NaCl. The results showed that when compared with the PMO treatment, salt stress significantly decreased the germination rate, fresh weight, carbohydrate content, and antioxidant enzyme activity of soybean and significantly increased the contents of reactive oxygen species, malondialdehyde, and osmoregulatory substances. However, PMO treatment enhanced the antioxidant defense system and significantly reduced the malondialdehyde content of soybean. Moreover, the activities of H^+^-ATPase and Ca^2+^-ATPase were significantly higher in treated soybean than in the control, and the content of ATP was also higher in treated soybean than in the control. Generally, PMO regulates the homeostasis of reactive oxygen species and reduces ATP consumption, thereby improving the ability of soybeans to germinate under salt stress. This study provides new insights into how nanomaterials improve plant salt tolerance.

## 1. Introduction

The Soybean (*Glycine max* (L.) Merr.) is one of the important oilseed crops and protein sources for humans worldwide. Over the past decade in China, the area planted with soybean, as well as soybean productivity, increased by 44.3% and 59.8%, respectively, but China has only achieved 18.5% of the soybean production the country needs to achieve self-sufficiency [1]. Therefore, a continued need exists to increase soybean production. However, stresses such as salinity, drought, and heat limit the area available in China to plant and produce soybean [2]. To date, about 8.7% of all farmland worldwide is affected by salinity [3]. Salinity is one of the major abiotic stresses impairing seed germination in soybean, including affecting the germination rate, resulting in longer germination times [4], thus undoubtedly increasing the cost of soybean production.

Seed germination is the first step of agriculture production. Improved germination is necessary for achieving high yield. Salinity impairs seed germination mainly through changes in osmosis, Na^+^ poisoning, and an excessive accumulation of reactive oxygen species (ROS) [5]. Plants have developed many pathways to reduce salt stress, such as the development of an antioxidant enzyme system and Na^+^ transport or salt storage mechanisms, but these require a large amount of energy and will significantly impair seed growth and seedling development [6,7]. To improve seed germination under saline conditions, many studies have focused on agricultural practices. Seed pre-treatment is often widely adopted to manage problems with soil salinity, and it has been reported to enhance antioxidant enzyme activity, reduce lipid peroxidation, activate pre-germinative enzymes, and regulate osmosis [5,8,9]. Aside from treatment with insecticides or fertilizers, seeds can also be treated with some specific solutions, such as mannitol and polyethylene glycol, to activate pre-germinative metabolism [10,11].

Seed nano-soaking (soaking the seed in nanomaterials) is a new choice for seed treatment, and it has been reported to have been successfully applied to improve seed germination and seedling stress tolerance [12,13]. Nanoparticles (NPs) such as Ag, ZnO, CeO_2_, and Fe_3_O_4_ NPs have been investigated for enhancing seed germination and development through modulating enzyme activity, hormonal balance, and nutrient acquisition [14,15]. However, recently reported agricultural nanomaterials involve biosafety considerations; the purification and assessment of agricultural nanomaterials for biosafety will undoubtedly increase their cost and lengthen the achievement of a transformation period [16]. Manganese (Mn), an important element in plants that plays an important role in plant development, is known as a critical component of antioxidant enzymes and the plant photosynthetic system. Studies in plants have shown that poly (acrylic) acid-coated Mn_3_O_4_ (PMO) could reduce the excessive accumulation of ROS and enhance seedling tolerance to stress such as that caused by salinity and drought [7,17]. Further study has shown that after the scavenging of ROS, PMO could modulate cytosol K^+^ retention and Na^+^ storage in vacuoles and enhance salt tolerance in cotton. Another study indicated that PMO enhanced the Na^+^ efflux to the apoplast in rapeseed [18]. Thus, PMO may exhibit different bioeffects on different species. It is surprising that little research has been conducted on the use of PMO to enhance abiotic stress tolerance in crop seed. To date, only one study has reported that PMO-treated maize seed has a relatively strong drought tolerance [17]. No known research has focused on PMO that was tested on soybean, so it remains unknown whether PMO nano-soaking would improve salt tolerance in soybean; the related mechanisms are still unknown.

Carbohydrate decomposition is an important part of seed germination and always involves energy metabolism [9]. Specifically, under saline soil conditions, knowing whether energy is used to combat salinity or is needed for seed development is important for seed germination. The production of soluble sugars and ATP has a positive relationship with seed germination [9]. An increase in the concentration of soluble sugar and ATP is known to enhance plant tolerance to salinity. The underlying mechanisms such as how PMO modulates soybean ROS homeostasis, carbohydrate decomposition, and energy metabolism are ambiguous. The role of PMO nano-soaking in the soluble sugar content and ATPase activities has been largely overlooked. The present study tries to address the above-mentioned problems by investigating the mechanisms regarding PMO-enhanced salt tolerance in soybean.

In this work, the application of PMO was studied with the goal of enhancing soybean germination. The level of ROS and the activities of antioxidant enzymes were studied in soybean with PMO soaking. Furthermore, the carbohydrate concentration in PMO-treated soybean seed and that in the control were compared under salinity stress. The concentration of ATP and the activities of H^+^-ATPase and Ca^2+^-ATPase were compared in salt-stressed soybean seeds with or without PMO nano-soaking. The results provide more knowledge about how the application of nanomaterials can enhance plant salt tolerance.

## 2. Results

### 2.1. Characterization of PMO

**Figure 1:**
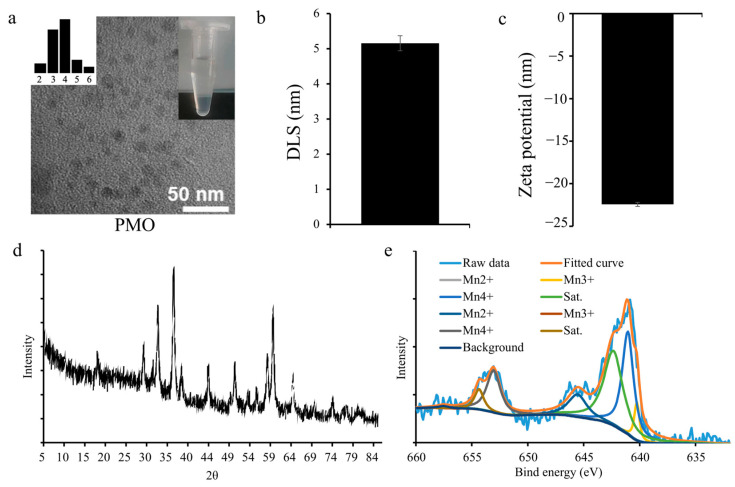
Characterization of PAA@Mn_3_O_4_ nanoparticles (PMO): (**a**) high resolution transmission electron microscopy (imaging of PMOs; (**b**) DLS size of PMOs; (**c**) zeta potential, mean ± SE (*n* = 4); (**d**,**e**) X-ray diffraction and X-ray photoelectron spectroscopy analysis of PMOs, respectively.

The synthesized PMO was characterized as shown in Figure 1. This TEM image shows the size and shape of Mn_3_O_4_ NPs, which were synthesized with the participation of PAA (PAA@Mn_3_O_4_-NPs, PMOs). These results showed that the PAA-modified Mn_3_O_4_ NPs (PMO) were spherical 0A with an average particle size of 5.15 ± 0.21 nm (Figure 1a,b). The potential of PMO was also measured, and those results showed that PMO was negatively charged, with a surface charge of −22.42 ± 0.24 mV (Figure 1c). XRD (X-ray diffraction) was used to determine the PMO element (Figure 1d). As the XRD result was consistent with the previous report [19], that PMO is Mn_3_O_4_. And the XPS result further found that there were three different Mn valences in the PMO surface, and its ratio is 1.3:1.3:1 for Mn^2+^:Mn^3+^:Mn^4+^, respectively.

### 2.2. Influence of PMO Soaking on Soybean Germination and Phenotype

**Figure 2:**
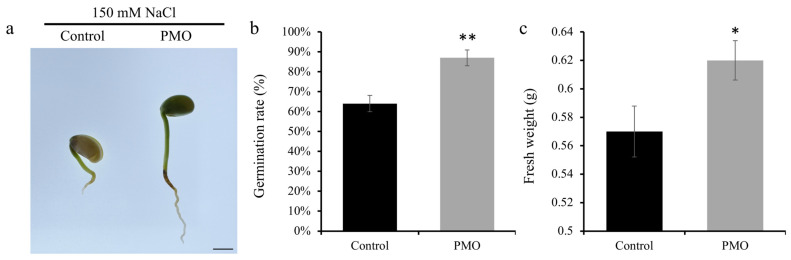
Phenotype of soybean treated with and without PMO under 7 days’ NaCl stress (150 mM). (**a**) Growth phenotype, scale bar = 1 cm; comparison between treatments was performed by independent samples t test (two tailed) for (**b**) germination rate (mean ± SE (*n* = 6)) and (**c**) fresh weight (mean ± SE (*n* = 20)). * and ** indicate significance at *p ≤* 0.05 and 0.01 levels, respectively. Note: PAA@Mn_3_O_4_ nanoparticles (PMOs).

We set up experiments with soybeans that were exposed to salt stress with or without PMO treatment to compare the effects of PMO on soybean germination under salt stress. First, the salt stress in the control group and PMO-treated soybean seedlings was explored by observing their growth phenotype and by measuring the germination rates and fresh weight of soybean seedlings. As shown in Figure 2b, the germination rate of soybean under PMO treatment was higher than that of soybean in the control group. Specifically, the PMO treatment significantly (*p* < 0.05) increased the germination rate of soybeans under salt stress. Furthermore, the PMO treatment also (*p* < 0.05) increased the fresh weight of the soybeans under salt stress, which was 9.9% higher when compared with the control group (Figure 2c), suggesting that PMO could improve salt tolerance during the germination and seedling-s stages in soybean.

### 2.3. PMO Modulates the Level of Osmotic Regulators in Soybean Seeds

**Figure 3:**
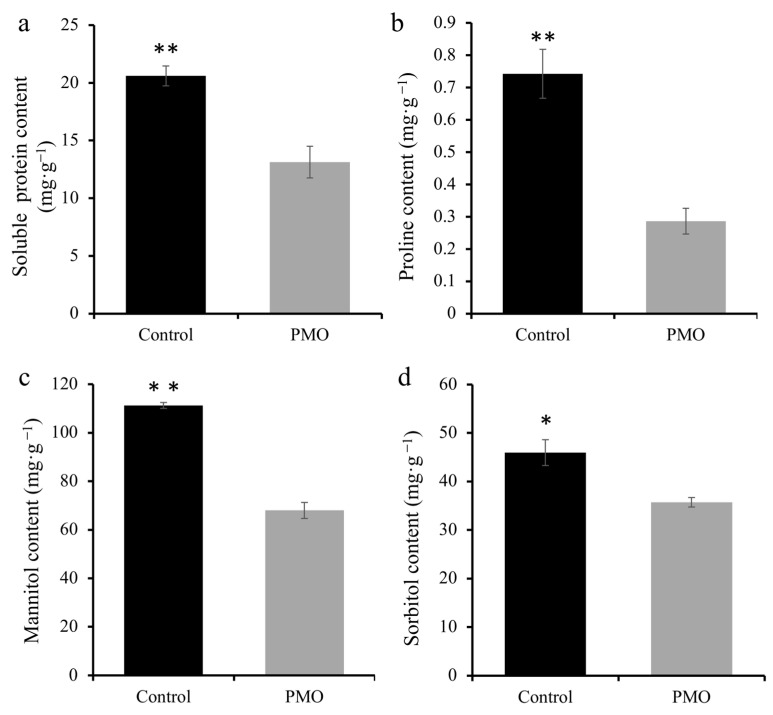
Osmoregulation substances content of soybean treated with and without PMO under 7 days’ NaCl stress (150 mM). (**a**) Soluble protein, (**b**) proline, (**c**) mannitol, and (**d**) sorbitol contents. Comparison between treatments was performed by independent samples t test (two tailed) in (**a**–**d**). * and ** indicate significance at *p ≤* 0.05 and 0.01 levels, respectively. Mean ± SE (*n* = 5). Note: PAA@Mn_3_O_4_ nanoparticles (PMO).

Plants tend to enhance their resistance to abiotic stress by increasing their content of osmoregulatory substances. Therefore, the content of soluble proteins, proline, mannitol, and sorbitol in soybeans under salt stress was evaluated; these substances are known to be osmoregulatory substances in plant cells [20]. However, it was found that the soluble protein content in soybeans under PMO treatment was significantly (*p* < 0.05) lower than that in the control group, with a decrease of 36.2% (Figure 3a). It can also be seen from Figure 3b that the proline content in soybeans under PMO treatment also decreased by 61.4% when compared with that in the control group. In addition, changes in the mannitol content in soybeans under PMO treatment are shown in Figure 3c. The mannitol content under PMO treatment was significantly (*p* < 0.05) lower than that of the control under salt stress, with a 38.8% decrease. Similarly, the sorbitol content was found to be significantly lower under the PMO treatment than that of the control (*p* < 0.05), which was a reduction of 22.3% (Figure 3d). In conclusion, PMO treatment led to a reduction in the levels of these osmoregulatory substances, rather than elevating their concentrations to improve plant salt tolerance.

### 2.4. PMO Nano-Soaking Enhanced Antioxidant Enzyme Activities to Modulate ROS Homeostasis

**Figure 4:**
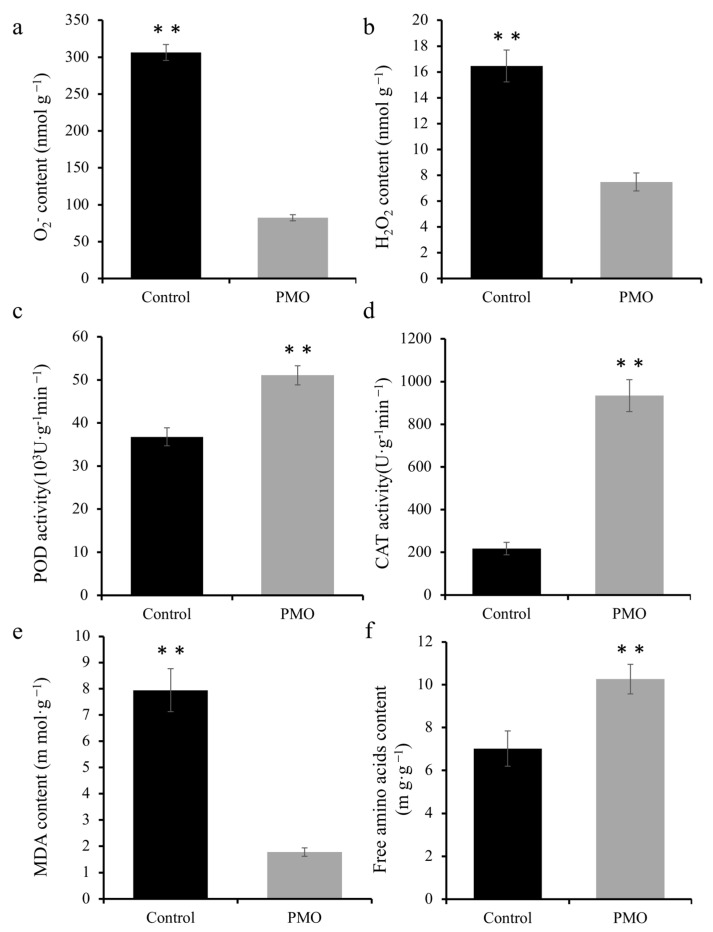
ROS content and anti-oxidase enzyme activities of soybean seeds with and without PMO treatment after 7 days’ NaCl stress (150 mM). (**a**) O_2_^−^ content, (**b**) H_2_O_2_ content, (**c**) peroxidase (POD) activity, (**d**) catalase (CAT) activity, (**e**) malondialdehyde (MDA) content, and (**f**) free amino acid content of soybean. A comparison between treatments was performed by independent sample t tests (two tailed) in (**a**–**f**). ** significance at *p ≤* 0.01 levels, respectively. Mean ± SE (*n* = 5).

Reactive oxygen species, such as O_2_^−^ and H_2_O_2_, produced by abiotic stress could cause oxidative stress; an accumulation of ROS oxidizes proteins, lipids, and DNA while damaging the cell membrane [6]. MDA (malondialdehyde) is the end product of lipid peroxidation, which reflects the antioxidant capacity and the degree of cell damage [4]. Thus, O_2_^−^, H_2_O_2_ and MDA levels in cells were detected during the present study. The results showed that when compared with control plants, the accumulation of O_2_^−^ and H_2_O_2_ under salt stress was reduced by 73.1% and 54.5% in the PMO-treated plants, respectively (Figure 4a,b). Meanwhile, under salt treatment, it was found that the MDA content in the PMO group was also approximately 77.5% lower than that of the control, which illustrated that the cell membrane was less damaged in the PMO group (Figure 4e). An MDA content increase typically implies peroxidative membranes. These results led to investigation of whether the activities of antioxidant enzymes were regulated by PMO NPs. Figure 4f shows that the free animo acid content increased by 46.1% in the PMO-treated group compared to the control group under salt stress. The reduction in free amino acid content may result from the oxidative degradation of proteins, which could also contribute to the observed decrease in ROS [21]. It is also worth noting that the activities of peroxidase (POD) and catalase (CAT) were also markedly elevated by 38.8% and 330%, respectively, in PMO plants when compared with non-treated plants (Figure 4c,d).

### 2.5. PMO Enhances the Carbohydrate Content in Salt Stressed Soybean Seeds

**Figure 5:**
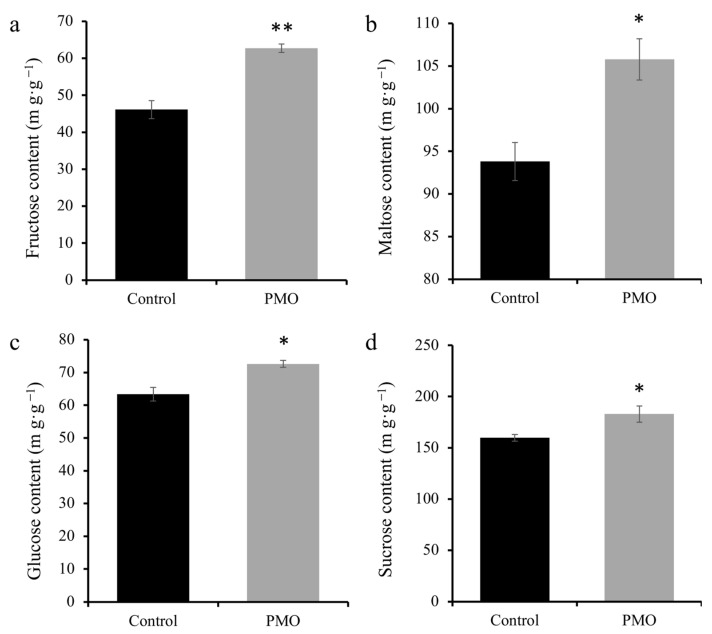
(**a**) Fructose, (**b**) maltose, (**c**) glucose, and (**d**) sucrose contents of soybean. The comparisons between treatments were performed by independent samples *t* test (two tailed) in (**a**–**d**). * and ** indicate significance at *p ≤* 0.05 and 0.01 levels, respectively. Mean ± SE (*n* = 5). Note: PAA@Mn_3_O_4_ nanoparticles (PMO).

Typically, the carbohydrate content of plants is likely to decrease when a plant is facing salt stress [22]. When PMO-treated plants were compared with control plants (Figure 5a,b), the fructose and maltose contents were likewise noticeably lower in the former and higher in the latter by 35.9% and 12.7%, respectively. Additionally, the contents of glucose and sucrose were both individually increased by about 14.6% and 14.5%, respectively (Figure 5c,d), suggesting that PMO-treated plants showed a higher salt tolerance than non-treated plants [23].

### 2.6. PMO Modulates H^+^-ATPase and Ca^2+^-ATPase Activities to Enhance ATP Content in Soybean Under Salt Stress

**Figure 6:**
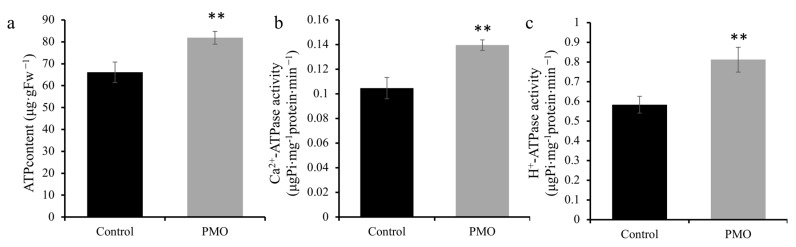
(**a**) ATP content, (**b**) Ca^2+^-ATPase activity, and (**c**) H^+^-ATPase activity in soybean. Comparisons between treatments were performed by independent samples *t* test (two tailed) in (**a**–**c**). ** indicate significance at *p ≤* 0.01 level. Mean ± SE (*n* = 6). Note: PAA@Mn_3_O_4_ nanoparticles (PMO).

Adenosine triphosphate is known to supply the energy required for various biological processes in plants, such as protein synthesis, DNA replication, and cell division. H^+^-ATPase can use the energy obtained from the hydrolysis of ATP to pump H^+^ out of the cytoplasm through the cell membrane [24]. Change in Ca^2+^-ATPase activities usually occur alone with different salt tolerance, so this plays an important role when the plant is under stress [25]. As such, the ATP content, as well as the activities of Ca^2+^-ATPase and H^+^-ATPase, was further investigated. As shown in Figure 6a, the ATP content of soybean under PMO treatment increased significantly (*p* < 0.05) by 23.8% when compared with the control. The impact of PMO treatment on Ca^2+^-ATPase and H^+^-ATPase activities is illustrated in Figure 6b,c. In comparison to the control group, PMO treatment led to significant (*p* < 0.05) increases in soybean Ca^2+^-ATPase and H^+^-ATPase activities under salt stress, with enhancements of 33.2% and 39.1% observed, respectively.

## 3. Discussion

### 3.1. PMO Modulates ROS Homeostats in Soybean Under Salt Stress

Salinity is well known as a major abiotic stress that reduces crop yield; soybean is sensitive to salinity. In terms of agricultural nano-technology, the use of the nano-soaking technique may be a good selection designed to enhance plant salt tolerance [12,13]. Regarding the cultivation of soybeans under salt stress, particularly in arid and semi-arid regions (such as Xinjiang), we compared the physiological alterations of soybeans subjected to salt stress with or without PMO treatment. The results of the present study showed that PMO enhanced soybean germination performance and fresh weight, while PMO nano-soaking significantly improved soybean tolerance to salinity. Similarly, previous studies have shown that the treatment of cotton and rapeseed with poly (acrylic) acid-coated cerium oxide nanoparticles (PNC) resulted in an increase in fresh weight [7,9]. Under salinity, a significant difference in the seed germination rate of soybean was observed between the PMO-treated and non-PMO-treated soybean plants at day 7. However, under salinity, PNC-treated cotton seeds did not exhibit a higher germination rate than those of the control group, although PNC has been shown to improve the germination rate in rapeseed [9,26]. These findings further suggest that in terms of the germination time and seed germination rate, factors such as the type of agricultural nanomaterial and plant species affect the outcome of seed nano-soaking.

An excessive accumulation of ROS is the major negative effect factor in plants under salinity. Previous studies have shown that seeds treated with nanomaterials accumulated ROS during the early part of the germination period. For example, PNC induced ROS accumulation in rapeseed and an increase in the concentration of active α-amylase [18]. The same results were reported in rice treated with cerium oxide nanoparticles and selenium-doped carbon dots [27]. Meanwhile, an excessive accumulation of ROS may induce toxic effects in plants. In addition, PMOs have been reported as having ROS scavengers [19]. In the present study, the control group had higher ROS (H_2_O_2_ and O_2_^−^) and MDA content than the PMO-treated seeds after 7 days of salinity stress, This is 73.1%, 54.5% and 77.5% higher respectively. After 7 days of salinity stress, POD and CAT activities were higher in PMO-treated seeds than in the control group. Previous studies have shown that PNC-treated rapeseed exhibited a significant increase in ROS after 1 h of priming and a decrease in ROS after 8 h of priming [18]. Researchers believed that the higher ROS content at 1 h of priming could be related to increases in the absorption of water and distribution of nanomaterials. At 8 h of priming, a decrease in ROS content was caused by the higher levels of SOD and POD activity. Early ROS accumulation may benefit seed germination and the activities of antioxidant enzymes. Similarly, the activities of SOD and POD have been reported to increase after treatment with nanomaterials, thereby controlling the excessive accumulation of H_2_O_2_ and O_2_^−^ in rapeseed, peas, and rice [9,28,29]. Interestingly, the content of ROS and antioxidants showed similar trends in the present seedling experiment. Foliar spraying of PMO has been shown to significantly reduce the excessive accumulation of ROS (H_2_O_2_ and O_2_^−^) in cotton leaf and to enhance SOD and POD activities under 200 mM NaCl stress, suggesting that PMO helped the plants to maintain ROS homeostasis in the plant cell [19]. The successful scavenging of ROS by PMO under NaCl stress has also been reported in maize [17].

### 3.2. PMO Reduces Osmotic Regulator Synthesis and Enhances Carbohydrate Degradation to Increase ATP Generation

**Figure 7:**
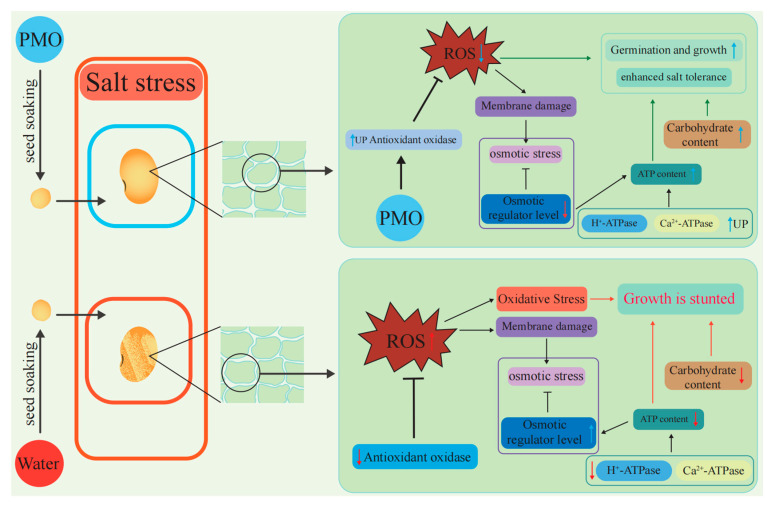
Diagram showing how reactive oxygen species (ROS) homeostasis is maintained and ATP consumption is reduced in the mechanism for PAA@Mn_3_O_4_ nanoparticle (PMO) nano-soaking to generate improved salt tolerance in soybean.

Besides excessive ROS accumulation, salinity also causes osmotic stress in plants. The ability of plants to maintain osmotic balance and to retain water is a critical factor for their salt tolerance [20]. To reduce the negative effects from osmosis, plants usually synthesize an osmotic regulator [30]. To our surprise, in the present study, after 7 days of salinity stress, the mannitol, sorbitol, proline, and protein content decreased. Reducing the synthesis of an osmotic regulator could save a plant a considerable amount of energy. Actually, PMO-treated soybean seeds had higher fructose, maltose, glucose, and sucrose content than the control group. A plant-synthesized osmotic regulator can reduce the toxic effects caused by osmotic stress, but this requires a large amount of ATP [31]. Thus, under salt stress, carbohydrate degradation is associated with plant salt stress tolerance. Mohammad et al. [32] reported that PNC could scavenge excessive accumulated ROS, enhance α-amylase activities, and improve K^+^ retention. It is well known that K^+^ is the key element for ATPase activities. In our study, both H+-ATPase and Ca^2+^-ATPase activities were enhanced in soybean, but the ATP content also increased. This could be related to a lower level of osmotic regulator synthesis and a higher soluble sugar concentration. Enhanced activity of H^+^-ATPase and Ca^2+^-ATPase is typically observed in plants exhibiting greater salt tolerance [33,34]. Salt stress leads to ATPase inactivity in the cell membrane [35]. The elevation of ATP content may be attributed to the effects of PMO; however, the specific mechanisms warrant further investigation. With PMO-maintained ROS homeostasis in soybean under salt stress, H+-ATPase and Ca^2+^-ATPase activities were obviously higher than those in the control group. Thus, maintaining ROS homeostasis and reducing ATP cost could be the other mechanisms underlying the improved salt tolerance observed with PMO nano-soaking in soybean (Figure 7).

### 3.3. Nano-Soaking Can Be Used to Enhance Plant Salt Tolerance

Various strategies, such as the application of organic fertilizer and the flooding of soil to remove salt, can improve the productivity of lands affected by salinity. However, water is a worthy source in some semiarid areas, making approaches expensive. Also, screening and breeding of salt-tolerant varieties of plants and the use of beneficial soil microorganisms could provide other options, but they are time-intensive. Based on this background, the use of nanobiotechnology is encouraged to address salt stress in soybean production.

Nanobiotechnology may provide a potentially effective solution to agriculture-related problems, especially salinity. For example, both PNC and PMO have been reported to improve cotton and rapeseed salt tolerance [7,13,19]. The first location in the interaction between nanomaterials and plants is in the plant cell. The cell wall and membrane will hinder the transfer of nanomaterials into plant cells. The cell wall has been reported to have pores and a negative charge. Normally, a particle with a size smaller than the pore’s diameter and with a positive potential can cross the cell wall relatively easily [36]. The different sizes and potentials of nanomaterials will affect how they enter plant cells. By exploring different leaf structures, researchers have found that the transfer of nanomaterials is dependent on their having a suitable size for cell wall transfer. However, in both cotton and maize, nanomaterials with positive potential have been shown to exhibit a better delivery efficiency [7,17,19]. Problems related to the transfer for nanomaterials usually have a secondary solution. The cell wall consists of pectin and cellulose; thus, cellulase can damage the cell wall and enable the entry of nanomaterials into the cell. Another important issue to be considered is that of biosafety. Recently, PMO has been found to be a biosafe nanomaterial because this kind of agricultural nanomaterial is suitable for use in the production of food crops.

## 4. Materials and Methods

### 4.1. Synthesis and Characterization of PMOs

**Figure 8:**
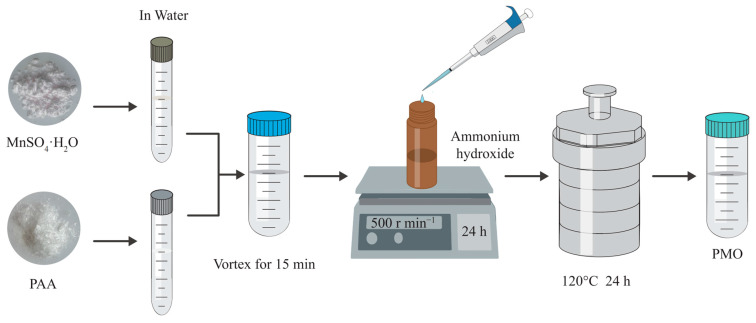
Schematic diagram of the synthesis of PAA (poly acrylic acid) @Mn_3_O_4_ nanoparticles (PMO).

Following our previous paper [19], the following formulations of PMO were synthesized (Figure 8).

MnSO_4_·H_2_O + 2NH_3_·H_2_O → Mn(OH)_2_ + (NH_4_)_2_SO_4_ + H_2_O (1)

Mn(OH)_2_ → MnO + H_2_O (2)

MnO + O_2_ → MnO_2_ (3)

2MnO + MnO_2_ → Mn_3_O_4_ (4)

The PMO materials were synthesized as follows. First, 0.425 g MnSO_4_·H_2_O (Guoyao, Co., Ltd., Shanghai, China) and 4.5 g poly (acrylic acid) (MW 1800, Sigma-Aldrich, St. Louis, MO, USA) were dissolved in 2.5 mL and 5 mL deionized water, respectively. The two solutions were mixed thoroughly at 2500 rpm for 15 min using a vortex mixer. The resulting mixture was then added dropwise to 15 mL of ammonium hydroxide solution (30%, Sigma-Aldrich) in a 30-mL brown vial; the reaction mixture was stirred constantly at 500 rpm for 24 h at 25 °C. Then, the solution was added into a 50-mL Teflon-equipped stainless autoclave and heated at 120 °C for 24 h. Then, the brown solution was centrifuged at 6000 rpm for 1 h to remove any debris and large agglomerates. The supernatant was further purified by a dialysis bag (MW 10000, Xi’an Youbo Biotechnology Co., Ltd., Xi’an, China) for 24 h, with the deionized water refreshed once every 8 h. The final concentration of PMO was calculated by the freeze-drying method. The final product was stored at 4 °C in a refrigerator for further use.

High-resolution transmission electron microscopy (HR-TEM) images were captured on a JEM-2100Plus (JEOL Co., Ltd., Tokyo, Japan) microscope with an accelerating voltage of 200 kV. Transmission electron microscopy (TEM) sampling was carried out by adding prepared samples dropwise to a copper mesh. The averaged TEM size of PMOs was obtained by counting and averaging the size of every individual NP in each TEM image. About 30–50 individual NPs were counted in each image. As described elsewhere, X-ray photoelectron spectroscopy (XPS) and X-ray diffraction (XRD) were conducted using a Thermo Scientific K-Alpha spectroscope (Thermo-Fisher, Waltham, MA, USA) and a Bruker D8 Advance 25 XRD instrument (Bruker, Germany) at Shiyanjia Lab (www.shiyanjia.com, Wuhan, China). The size and positive zeta potential of NPs were characterized by a model Nano 90 Malvern zetasizer (Malvern, UK).

### 4.2. Seed Materials, Seed Soaking, Stress Treatments, and Growth Conditions

**Figure 9:**
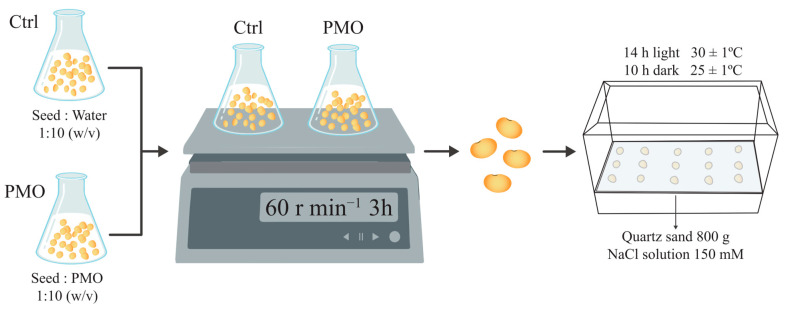
Schematic diagram of the experiment. Note: PAA@Mn_3_O_4_ nanoparticles (PMO).

Seeds of soybean variety Sui nong 35 (SN35) were used in this experiment. First, 100 mg/L PMO was used as a soaking agent in this experiment. Seeds were immersed in PMO and, for the control, in water. The conical flasks containing the seeds and soaking solution were placed on a mechanical shaker (60 rpm) under dark conditions with constant gentle agitation for 3 h, with a seed to solution ratio of 1:10 (*w*/*v*). After 3 h of priming, the soaked seeds were sown in plastic boxes (19 cm × 13.5 cm × 8 cm). Each box contained 800 g quartz sand and 300 mL of 150 mM NaCl solution. Fifteen seeds were sown evenly in each box, and the boxes were then exposed to light for 14 h and placed in the dark for 10 h, with temperatures of 30 ± 1 °C and 25 ± 1 °C, respectively (Figure 9). The germination trial was terminated 7 days after salt treatment. Then, the biomass of the seeds was recorded immediately along with the germination rate.

### 4.3. Determination of ROS Content, Antioxidant Enzyme Activity, and Malondialdehyde (MDA) Content

The hydrogen peroxide (H_2_O_2_) and O_2_^−^ contents in soybean were measured using ROS assay kits as follows. Briefly, an H_2_O_2_ assay kit (Nanjing Jiancheng Biotechnology Co., Ltd., Nanjing, China) was used to determine the H_2_O_2_ content of soybean seedlings. For the determination of the superoxide anion (O_2_^−^), an “O_2_^−^ assay kit” (Beijing Leigen Biotechnology Co., Ltd., Beijing, China) was used. Peroxidase (POD), catalase (CAT), and MDA assay kits (Solarbio Biotech Co., Ltd., Beijing, China) were used to determine POD, CAT, and MDA activity, respectively. These measurements were carried out according to the instructions provided by the manufacturer.

### 4.4. Measurement of Soluble Protein, Free Amino Acid, Proline, and Soluble Sugar Concentrations

The soluble protein concentration of each sample was determined using brilliant blue G-250 regent with bovine serum albumin as a standard according to Bradford [37]. Concentrations of free amino acid and proline were analyzed by the methods described by Živanović et al. [38]. Frozen tissues (0.5 g) were homogenized in 10 mL of 3% aqueous sulfosalicylic acid. After filtering the homogenate, 2 mL of filtrate was mixed with 2 mL of glacial acetic acid and 2 mL of acidic ninhydrin. The reaction mixture was incubated at 100 °C for 1 h and then cooled on ice for 20 min before extraction with 4 mL of toluene. The concentrations of free amino acids and proline were measured with a spectrophotometer (Shimadzu Co., Ltd., Kyoto, Japan) at A580 nm and A520 nm, respectively. The total soluble sugar concentration was determined by the methods described by Du et al. [39]. Tissues (0.1 g) were added to 5 mL of 80% (*v*/*v*) ethanol and incubated in a water bath at 80 °C for 30 min, followed by centrifugation at 10,000× *g* for 10 min. The residue was extracted two more times using 80% ethanol. The three supernatants were combined, and 80% ethanol was added to a total volume of 5 mL. The soluble sugar concentration was determined spectrophotometrically at an A620 nm wavelength.

### 4.5. Determination of Carbohydrate Contents

For measurement of the content of the four main soluble sugar species, sucrose, glucose, maltose, and fructose, sugars were extracted from 25 mg of leaf tissues in 1 mL of distilled water by sonication at 4 °C for 10 min, and the extract was centrifuged at 12,000× *g* for 15 min at 4 °C. This step was repeated twice, after which the supernatants were collected in a 2-mL centrifuge tube. Sugars were enriched from the crude extracts by solid phase extraction using a C18 solid phase extraction cartridge (Waters, Milford, MA, USA). Subsequently, 1 mL of each sample was run through a 0.22 µm Sep-Pak filter (Waters). High performance liquid chromatography (Shimadzu Co., Ltd.) was performed by injecting 5 μL of each filtered sample into a Sugar-Pak1 column (6.5 mm × 300 mm, Waters), running it over 30 min at a flow rate of 0.6 mL∙min^−1^, and monitoring the eluents with a UV detector at 210 nm. The temperatures of the column and reference cells were maintained at 85 °C and 35 °C, respectively.

### 4.6. Content of Mannitol and Sorbitol

To determine the content of mannitol and sorbitol in soybean seeds, 100 mg of each dried sample was placed into a centrifuge tube and mannitol and sorbitol were extracted with 4 mL of 80% alcohol; samples were then placed into a boiling water bath for 1 h and then centrifuged at 3000× *g* for 15 min, and the supernatant was collected. Next, the samples were re-expounded twice, the supernatant was combined three times, and the volume was brought to 10 mL for later use. The mannitol and sorbitol concentrations were determined by high performance liquid chromatography using an LC 10AVP chromatograph (Shimadzu Co., Ltd.). The column was a sugar PAK 1 (7.8 mm × 300 mm); the mobile phase was PBS (0.1 M, pH 7. 0)-acetonitrile (82:18, *v*:*v*); the flow rate was 1.0 mL min^−1^; the injection volume was 20 μL; and an SPD-10AVD ultraviolet light detector (Shimadzu Co., Ltd.) with a detection wavelength of 245 nm was used.

### 4.7. Quantification of ATP Levels and Ca^2+^-ATPase and H^+^-ATPase Assay

The ATP content of each sample was determined using tissue samples of the control and PMO. The homogenate was centrifuged at 8000× *g* at 4 °C for 10 min. Absorbance of the collected supernatant was measured at 700 nm with a UV spectrophotometer (Shimadzu Co., Ltd.). The ATP was measured using an ATP Content Assay Kit (Solarbio Biotech Co., Ltd.).

The Ca^2+^-ATPase activity on the plasma membrane was determined according to the method of Liang et al. [40], with some modifications. The reaction was initiated by adding 50 µL of 30 mM ATP-Na_2_ and 50 µL extracted protein (plasma membrane) to reaction reagents and reacted in water bath (30 °C) for 20 min. The reaction was terminated by adding 50 µL of 55% TCA. The absorbance was recorded at 660 nm by using a spectrophotometer (Shimadzu Co., Ltd.). The Ca^2+^-ATPase activity was determined by measuring the amount of Pi released in the absence and the presence of 3 mM CaCl_2_ in the reaction solution. The reaction reagents (0.5 mL, pH 7.5) contained 25 mM HEPES, 1 mM EGTA, 1 mM NaN_3_, 50 mM NaNO_3_, 50 mM KCl, 8 mM MgSO_4_, and 80 μM Na_2_MoO_4_; the pH of the reaction reagents was adjusted by 0.5 M Tris.

Plasma membrane vesicles were isolated from the soybean by two-phase partitioning according to the method described by Larsson et al. and by Liang et al. [41]. The concentration of proteins was measured according to the method described by Bradford [37]. The purity of the plasma membrane obtained was determined by analyzing the H^+^-ATPase hydrolytic activity of the membrane fraction in the presence and absence of inhibitors [42]. The plasma membrane H^+^-ATPase activity was determined by measuring the Pi amount after 30 min of the hydrolysis reaction according to the method described by Liang et al. [41].

### 4.8. Statistical Analysis

All data (mean ± SD, *n* = biological replicates) were analyzed using SPSS 29.0 (SPSS Inc., Chicago, IL, USA). A comparison between treatments was performed by independent samples *t* tests (two-tailed) or one-way analysis of variance based on Duncan’s multiple range test (two-tailed). The significance levels were * *p* < 0.05 and ** *p* < 0.01.

## 5. Conclusions

In summary, this study showed for the first time that the germination rate, fresh weight, and carbohydrate content of soybean (including fructose, maltose, glucose, and sucrose) during germination under 150 mM NaCl was significantly reduced compared with that under PMO treatment. In addition, the aggravated membrane damage caused by the accumulation of ROS caused soybean to consume more ATP in order to synthesize osmoregulatory substances such as soluble protein, proline, mannitol, and sorbitol to alleviate the osmotic stress caused by salt. The PMO treatment increased the activity of antioxidant enzymes (CAT, POD), decreased the accumulation of ROS, and significantly decreased the content of MDA. Moreover, the activities of H^+^-ATPase and Ca^2+^-ATPase were significantly increased under PMO treatment when compared with the control, and the content of ATP was also higher than that of the control. These results suggest that maintaining ROS homeostasis and reducing ATP consumption may be the mechanisms by which PMO nano-soaking improved the salt tolerance in soybean.

## Data Availability

All data from this study are available in this article.
